# α-Lipoic Acid Ameliorates The Changes in Prooxidant-Antioxidant Balance in Liver and Brain Tissues of Propylthiouracil-Induced Hypothyroid Rats 

**DOI:** 10.22074/cellj.2020.7049

**Published:** 2020-09-08

**Authors:** Adile Merve Baki, Abdurrahman Fatih Aydın, Pervin Vural, Vakur Olgaç, Semra Doğru Abbasoğlu, Müjdat Uysal

**Affiliations:** 1. Department of Biochemistry, Istanbul Faculty of Medicine, Istanbul University, Istanbul, Turkey; 2. Institute of Oncology, Department of Pathology, Istanbul University, Istanbul,Turkey

**Keywords:** Brain, Hypothyroidism, Lipoic Acid, Liver, Prooxidant-Antioxidant Balance

## Abstract

**Objective:**

There are controversial data about the prooxidant-antioxidant balance in hypothyroidism. We aimed to investigate the effect of α-lipoic acid (ALA) on oxidative stress parameters in the liver and brain of propylthiouracil (PTU)-induced hypothyroid rats.

**Materials and Methods:**

In this experimental study, PTU (500 mg/L) was given to rats in drinking water for 10 weeks. ALA (0.2% in diet) alone and together with thyroxine (T4, 20 µg/kg body weight, s.c) were given to hypothyroid rats in the last 5 weeks of the experimental period. The levels of reactive oxygen species, malondialdehyde, protein carbonyl, ferric reducing antioxidant power (FRAP) and glutathione (GSH) levels, superoxide dismutase, and GSH peroxidase activities were determined in the liver and brain of rats. Histopathological examinations were also performed.

**Results:**

Prooxidant parameters were increased in the brain but not liver in hypothyroid rats. ALA treatment alone lowered enhanced brain oxidative stress in hypothyroid rats. Also, ALA was found to ameliorate the changes as a result of oxidative stress arising from T4 replacement therapy.

**Conclusion:**

Our results indicate that ALA alone and together with T4 may be useful in reducing oxidative stress in thyroid dysfunctions.

## Introduction

Thyroid hormones (THs) (thyroxine and triiodothyronine, T4, and T3) are necessary for various physiological functions such as growth, development, and reproduction, and they regulate lipid and carbohydrate metabolism (1). They control the body’s metabolism rate by regulating the rate of tissue oxygen consumption. Therefore, hyperthyroidism is characterized by accentuated oxidative metabolism and reactive oxygen species (ROS) production leading to tissue injury (2). Furthermore, THs control protein, vitamin, and antioxidant enzyme production, and breakdown (3, 4).

Hypothyroidism is a pathological condition related to the hypometabolic state, decreased mitochondrial oxygen utilization, low tissue proliferation, and reduction of ROS formation (5-7). Moreover, many experimental (8-10) and clinical (11, 12) studies showed that hypothyroidism is also related to increased ROS production and oxidative stress in several tissues, as observed in hyperthyroidism. However, the mechanisms are different in two clinical situations. Increased oxidative stress was attributed to the decrease in antioxidant levels and an increase in atherogenic lipids providing a substrate for lipid peroxidation in hypothyroidism (3, 13).

Several studies reported that antioxidant therapy may help prevent the oxidative stress seen in hypothyroidism and may provide support to the conventional L-thyroxine treatment. For this purpose, various antioxidants such as vitamin E, curcumin, and taurine have been used in hypothyroidism, and some favorable results have been obtained (8, 14, 15). α-Lipoic acid (ALA) is a mitochondrial coenzyme with significant antioxidant properties. ALA supports the regeneration of many antioxidants such as vitamin E and C, glutathione (GSH), and coenzyme Q10, repairs oxidized proteins, creates complexes with metal ions such as copper, manganese, and zinc, and prevents the formation of ROS (16). Indeed, it has been reported that ALA possesses beneficial effects on various conditions related to increased oxidative stress (16, 17).

In this study, we wanted to investigate prooxidantantioxidant balance in liver and brain tissues before and after ALA and/or L-thyroxine (T4) replacement therapy changes in a 6-propyl-2-thiouracil (PTU)- induced hypothyroid rat model. Besides, the determinations of some biochemical indicators in serum and histopathological observations in examined tissues were performed.

## Materials and Methods

In this experimental study, Sprague-Dawley albino male rats (weighing 250-350 g), purchased from the Institute of Experimental Medicine of Istanbul University, Turkey, were housed in ordinary metallic cages in a room with the temperature regulated at 21 ± 1°C and light/ dark cycles (12 hours). The experimental procedure met the guidelines of the Institutional Animal Care and Use Committee of Istanbul University (Project No. 2014/111). Used chemicals were obtained from Sigma (Sigma Chemical Co., St. Louis, MO, USA).

Animals were divided into six groups as control, ALA, PTU, PTU+ALA, PTU+T4, and PTU+T4+ALA; each group included 6 rats. Control group: rats were fed with a standard diet and drinking water ad libitum for 10 weeks.

ALA group: rats were fed initially with the standard diet for 5 weeks. For next 5 weeks, rats were fed with ALA (0.2%, w/w) supplemented diet. The utilization of ALA by rats was approximately equivalent to 100 mg/kg body weight/day. Tap water was given as drinking water in this group.

Other groups were fed standard diet and treated with PTU (500 mg/L, w/v) in drinking water for 5 weeks. After 5 weeks, the administration of PTU was continued for another 5-week period in these groups. Rats of PTU and PTU+T4 groups were fed standard diet, while rats of PTU+ALA and PTU+T4+ALA groups were fed with 0.2% (w/w) ALA containing diet for the last 5 weeks. Rats of PTU+T4 and PTU+T4+ALA groups were also treated with L-thyroxine (20 μg/kg/day, s.c.) in this period.

After 10 weeks of the experimental period, the blood samples were drawn by cardiac puncture under pentobarbital anesthesia (50 mg/kg, i.p.) following overnight fasting. Blood samples were centrifuged at 1500 x g for 10 min to obtain serum fraction. Liver and brain tissues were removed immediately after blood collection, rinsed with ice-cold saline and blotted with filter paper. Tissues were homogenized in ice-cold 0.15 M KCl (10%, w/v), and tissue homogenates were centrifuged at 600 x g for 10 minutes to remove crude fractions. In obtained supernatants, ROS, malondialdehyde (MDA), protein carbonyl (PC), ferric reducing antioxidant power (FRAP), and GSH determinations were performed. These samples were centrifuged at 10000 x g for 20 minutes to obtain the postmitochondrial fraction. This fraction was stored at -80°C for the analysis of superoxide dismutase (SOD) and glutathione peroxidase (GSH-Px) activities.

### Determinations in serum

Serum fT3 and fT4 were measured on the Elecsys autoanalyzer (Roche Diagnostics, Germany). Serum glucose, total cholesterol (TC), triglyceride (TG) and albumin levels, alanine aminotransferase (ALT) and aspartate aminotransferase (AST) activities were assayed on Cobas Integra 800 autoanalyzer (Roche Diagnostics, Germany).

### Determinations of reactive oxygen species formation, lipid peroxidation, and protein oxidation products

Ultraspec 3000 spectrophotometer (Pharmacia Biotech, Biochrom Ltd. Cambridge, UK) was used for the spectrophotometrical measurements. ROS formation was assayed fluorometrically (18). After 30 minutes of the incubation period with 100 μM 2,7-dichlorodihydrofluorescein diacetate (DCFH-DA), the fluorescence of formed product was read on a fluorometer (Fluoroskan Ascent FL, Thermo Scientific Inc, USA) (excitation- 485 nm, emission- 538 nm). Results were expressed as relative fluorescence units (RFU). Lipid peroxidation was examined spectrophotometrically using the reaction between MDA and thiobarbituric acid at 535 nm (19). The MDA concentrations of samples were calculated using an extinction coefficient of 1.56×10^5^ M^-1^cm^-1^. MDA levels were expressed as pmol MDA/ mg protein. The oxidative protein damage (PC levels) was measured by the quantification of carbonyl groups based on their reaction with 2,4-dinitrophenylhydrazine (DNPH). Calculation of results was performed using a molar absorption coefficient of 22,000 M^-1^cm^-1^ at 360 nm and expressed as nmol carbonyl per mg protein (20).

### Determinations of non-enzymatic and enzymatic antioxidants

Total antioxidant status was evaluated using FRAP assay (21). In this assay, a ferric- tripyridyltriazine (Fe^3+^- TPTZ) complex is reduced to the ferrous form, which can be monitored by measuring the change in absorbance at 593 nm. GSH levels were measured with 5,5-dithiobis- (2-nitrobenzoate) at 412 nm (22). The SOD activity was assayed by its ability to increase the effect of riboflavinsensitized photooxidation of o-dianisidine (23). GSH-Px activity (24) was assayed using cumene hydroperoxide as a substrate. Protein determination was performed using the bicinchoninic acid method (25).

### Histopathologic evaluation

Liver tissues were fixed in 10% buffered formaldehyde, processed, and stained with hematoxylin and eosin (H&E) for histopathologic examination.

### Statistical analyses

ANOVA (post hoc Tukey HSD) and Kruskal-Wallis (post hoc Mann-Whitney U) tests were used for statistical evaluation. A P<0.05 was considered to be statistically significant. All statistical analyses were performed with IBM SPSS Statistics for Windows (version 21, SPSS Inc., Chicago, IL, USA). The results were expressed as mean ± SD.

## Results

### Body weight, liver weight, and liver indices

Body and liver weights significantly decreased, while the liver index did not change in PTU and PTU+ALA groups. Giving of T4 to PTU-treated rats resulted in significant increases in body and liver weights and liver index as compared to the PTU group. These parameters remained unaltered in PTU+T4 rats due to ALA treatment (Table 1).

**Table 1:** Effects of ALA alone or together with T4 treatment on body and liver weights, liver index, serum glucose, TC, TG, albümin levels and ALT and AST activities of PTU administered rats


Parameters	Control	ALA	PTU	PTU+ALA	PTU+T4	PTU+T4+ALA

Body weight (g)	310.8 ± 38	319.5 ± 44.3	217.0 ± 9.32^a^	228.3 ± 6.28^a,b^	259.7 ± 24.5^a,b^	276.5 ± 18.1^b^
Liver weight (g)	7.94 ± 1.21	9.51 ± 1.90	5.32 ± 0.48^a^	5.31 ± 0.44^a^	7.36 ± 0.81^b^	8.43 ± 0.97^b^
Liver index^*^(%)	2.55 ± 0.16	2.95 ± 0.22^a^	2.45 ± 0.21	2.32 ± 0.14	2.84 ± 0.24^b^	3.04 ± 0.23^a,b^
Free T3 (pmol/L)	3.17 ± 0.40	3.56 ± 0.56	1.35 ± 0.30^a^	1.16 ± 0.13^a^	1.70 ± 0.16^a^	1.78 ± 0.15^a^
Free T4 (pmol/L)	26.3 ± 5.90	28.3 ± 5.76	1.75 ± 0.95^a^	0.98 ± 0.18^a^	48.1 ± 8.87^a,b^	43.6 ± 10.9^a,b^
Glucose (mg/dL)	139.0 ± 8.10	142.6 ± 11.8	103.6 ± 13.5^a^	102.0 ± 5.95^a^	129.6 ± 17.7^b^	133.6 ± 12.4^b^
TC (mg/dL)	56.1 ± 11.1	46.1 ± 6.68	80.7 ± 8.29^a^	79.3 ± 7.95^a^	58.0 ± 10.0^b^	49.1 ± 3.91^b^
TG (mg/dL)	48.6 ± 9.77	40.6 ± 14.3	28.1 ± 4.36^a^	29.3 ± 7.69^a^	28.1 ± 7.41^a^	18.5 ± 4.38^a^
Albumin (g/dL)	3.77 ± 0.28	3.69 ± 0.23	3.57 ± 0.21	3.60 ± 0.17	3.51 ± 0.21	3.68 ± 0.15
ALT (U/L)	44.6 ± 6.50	55.8 ± 16.7	62.8 ± 8.63	105.2 ± 21.2^a,b^	85.6 ± 36.3^a^	74.5 ± 17.1
AST (U/L)	117.0 ± 16.8	111.8 ± 16.9	118.5 ± 18.2	127.2 ± 21.3	180.0 ± 33.8^a,b^	145.1 ± 13.2^c^


Data are presentes as mean ± SD, n=6 each. ALA; α-lipoic acid, TC; Total cholesterol, TG; Triglyceride, ALT; Alanine aminotransferase, AST; Aspartate aminotransferase, PTU; Propylthiouracil, *; Liver weight×100/body weight,^a^; P<0.05 as compared with control,^ b^; P<0.05 as compared with PTU group, and ^c^; P<0.05 as compared with PTU+T4.

### Serum thyroid function tests

PTU administration significantly decreased serum fT3 and fT4 levels. ALA treatment did not change the levels of fT3 and fT4 in hypothyroid rats. When PTUtreated rats were administered with T4, serum fT4 levels increased significantly, while fT3 unchanged as compared to the PTU group. ALA treatment did not change fT3 and fT4 levels in rats of the PTU+T4 group (Table 1).

### Serum glucose, lipid profile parameters, and liver function analyses

Serum glucose and TG levels significantly decreased, TC levels significantly increased, while albumin levels, ALT, AST activities did not change in the PTUtreated group. ALA administration caused an increase in ALT activity in the PTU-treated group. However, other serum parameters did not alter. T4 administration to PTU-treated rats resulted in increased glucose levels and AST activity together with lowered TC levels, but TG and albumin levels and ALT activity remained unchanged. In the PTU+T4+ALA group, elevated serum AST activity was found to diminish as compared to the PTU+T4 group (Table 1).

### Prooxidant and antioxidant status in the liver tissue

**Fig.1:**
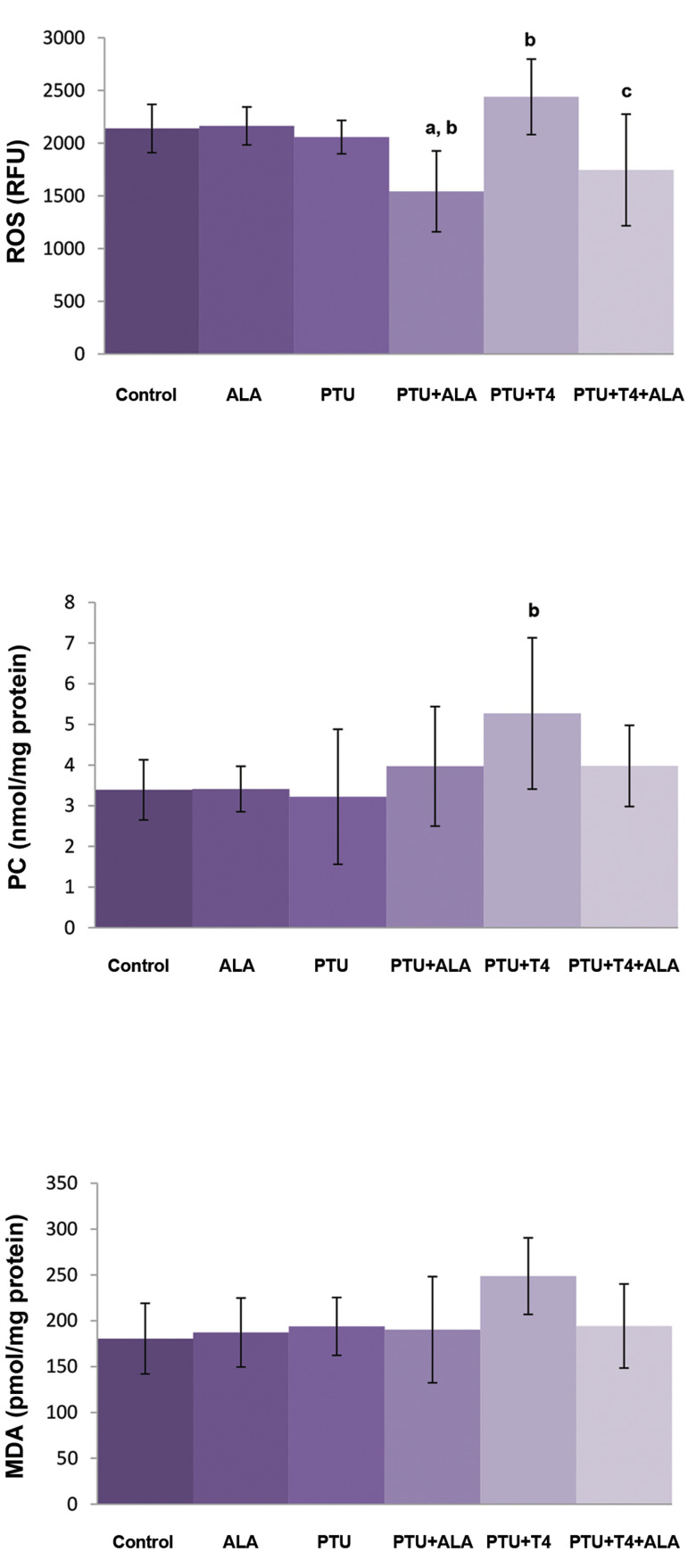
Effects of ALA alone or together with T4 treatment on liver ROS formation, MDA and PC levels in liver tissue of PTU administered rats (mean ± SD). ALA; α-lipoic acid, ROS; Reactive oxygen species, MDA; Malondialdehyde, PC; Protein carbonyl, PTU; Propylthiouracil, ^a^; P<0.05 compared with control,^b^; P<0.05 compared with PTU, and ^c^; P<0.05 compared with PTU+T4.

There were no changes in hepatic ROS formation, MDA, and PC levels in PTU-induced hypothyroid rats. ALA administration to PTU-treated rats (PTU+ALA) resulted in significant decreases in hepatic ROS formation, but MDA and PC levels did not alter when compared with the PTU group. Administration of T4 to the PTU-treated group resulted in significant increases in hepatic ROS formation (18.5%) and PC levels (63.7%) as compared to the PTU group. MDA levels were also increased (28.3%), but this increase was not significant. When ALA was administered to rats in the PTU+T4 group, hepatic ROS levels were found to decrease significantly. Hepatic MDA (21.9%) and PC (24.5%) levels were also decreased, but these decreases were not significant (Fig .1).

**Fig.2:**
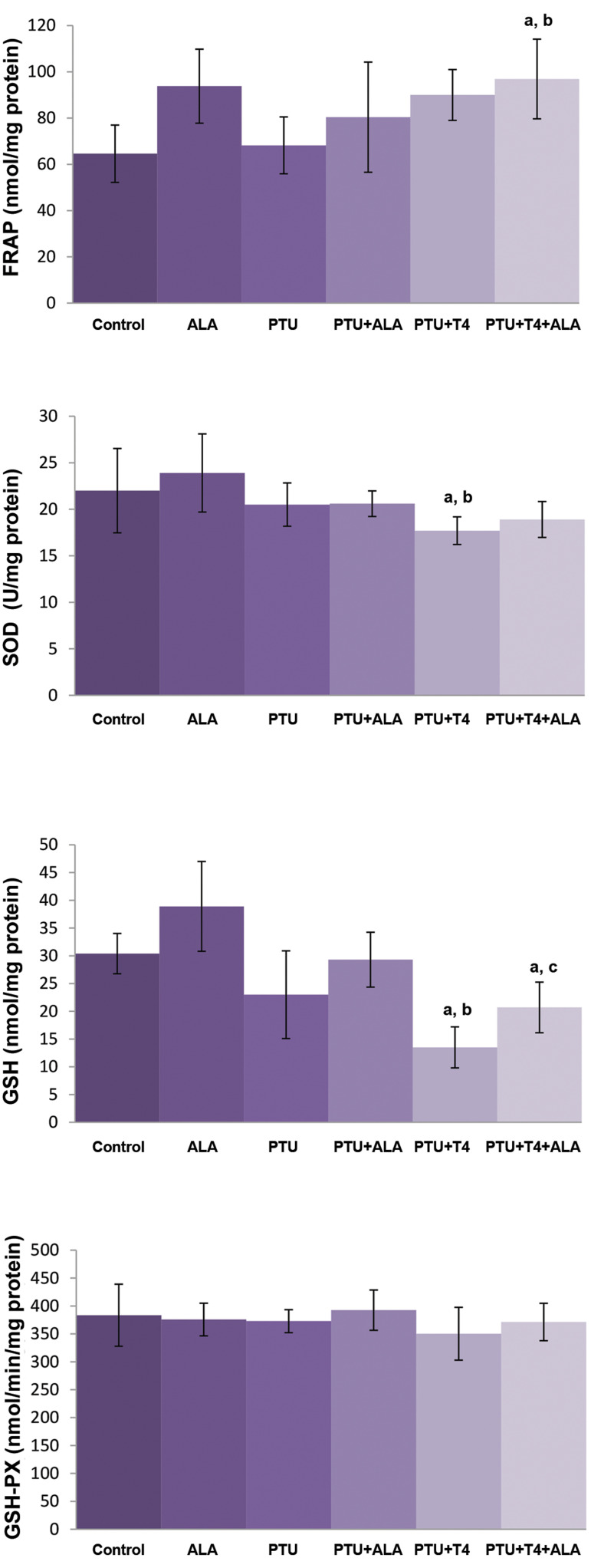
Effects of ALA alone or together with T4 treatment on liver FRAP and GSH levels, SOD and GSH-Px activities in liver tissue of PTU administered rats (mean ± SD). ALA; α-lipoic acid, FRAP; Ferric reducing antioxidant, GSH; Glutathione, SOD; Superoxide dismutase, GSH-Px; Glutathione peroxidase, PTU; Propylthiouracil, ^a^; P<0.05 compared with control,^b^; P<0.05 compared with PTU, and ^c^; P<0.05 compared with PTU+T4.

In PTU-treated rats, no changes were detected in hepatic FRAP and GSH levels, and SOD and GSHPx activities when compared with control rats. These parameters did not change due to ALA treatment. T4 administration to the PTU-treated group resulted in significant decreases in hepatic GSH levels and SOD activity, but FRAP levels and GSH-Px activity did not alter as compared to the PTU group. No changes were detected in FRAP levels, SOD and GSH-PX activities, but GSH levels increased in the liver of rats of the PTU+T4+ALA group as compared to the PTU+T4 group (Fig .2).

### Prooxidant and antioxidant status in the brain tissue

**Fig.3:**
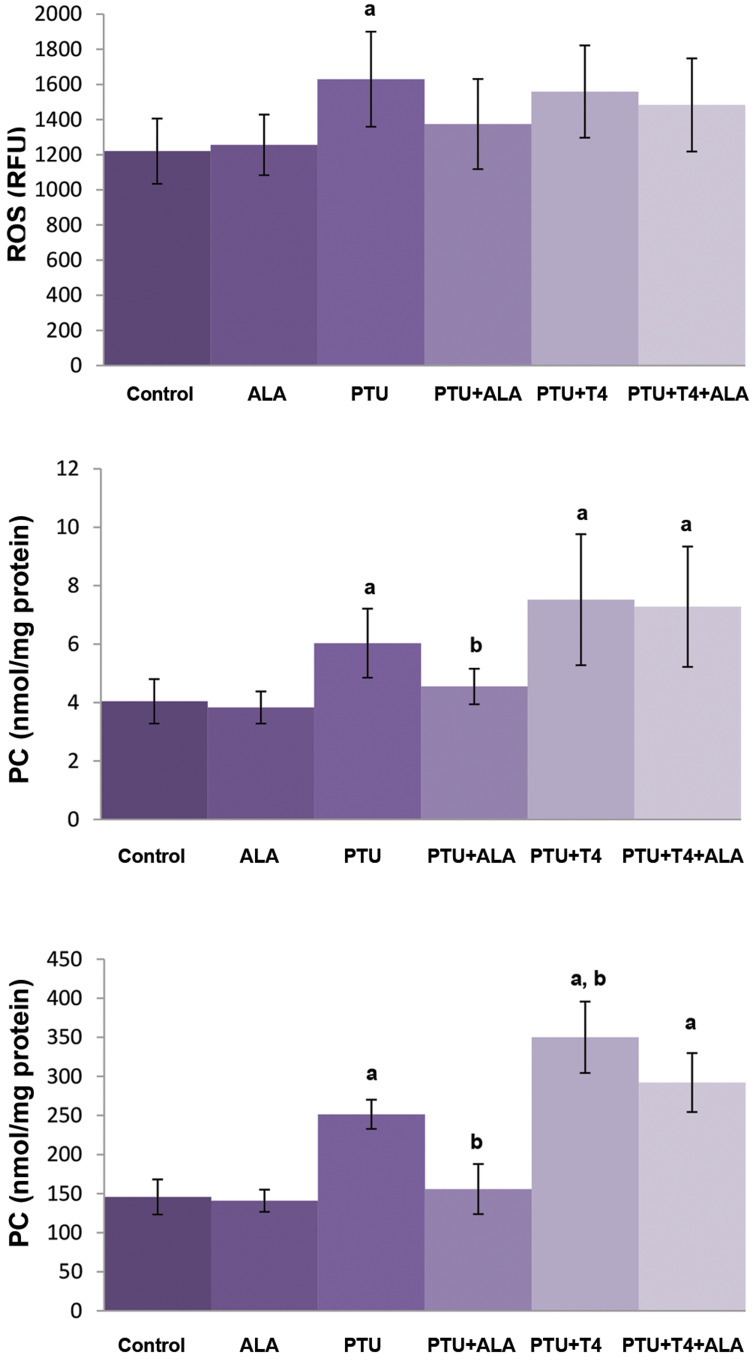
Effects of ALA alone or together with T4 treatment on brain ROS formation, MDA and PC levels in brain tissue of PTU administered rats (mean ± SD). ALA; α-lipoic acid, ROS; Reactive oxygen species, MDA; Malondialdehyde, PC; Protein carbonyl, PTU; Propylthiouracil, ^a^; P<0.05 compared with control, and ^b^; P<0.05 compared with PTU.

Brain ROS formation, MDA, and PC levels were found to increase in PTU-induced hypothyroid rats. ALA administration to the PTU-treated group (PTU+ALA) decreased MDA and PC levels, but not ROS formation as compared to the PTU group. Although T4 administration to PTU-treated rats caused further increases in brain MDA levels, ROS formation and PC levels did not alter as compared to PTU group. ALA administration to rats of PTU+T4 was found not to alter these parameters in the brain tissue (Fig .3).

**Fig.4:**
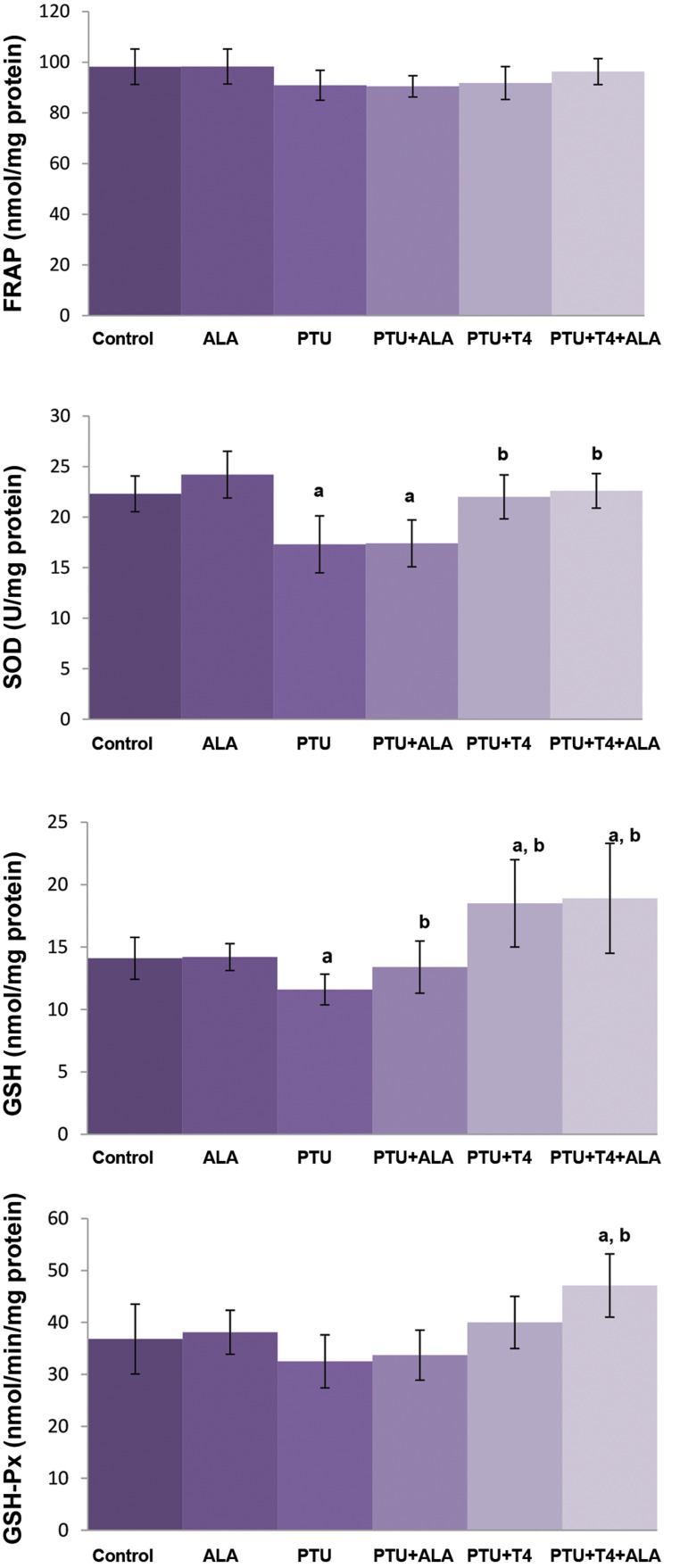
Effects of ALA alone or together with T4 treatment on brain FRAP and GSH levels, SOD and GSH-Px activities in liver tissue of PTU administered rats (mean ± SD). ALA; α-lipoic acid, FRAP; Ferric reducing antioxidant, GSH; Glutathione, SOD; Superoxide dismutase, GSH-Px; Glutathione peroxidase, PTU; Propylthiouracil, ^a^; P<0.05 compared with control, and^ b^; P<0.05 compared with PTU.

GSH levels and SOD activity decreased, but FRAP levels and GSH-Px activity did not alter in the brain tissue of PTU-treated rats as compared to the cotrol group. Increases in GSH levels were detected, but FRAP levels, SOD, and GSH-Px activities remained unchanged in the PTU-treated group due to ALA treatment. In PTU+T4 group, brain GSH levels and SOD activity increased, but FRAP levels and GSH-Px activity did not alter as compared to the PTU group. There was no difference in antioxidant parameters between PTU+T4 and PTU+T4+ALA groups (Fig .4).

### Histopathological evaluation

**Fig.5:**
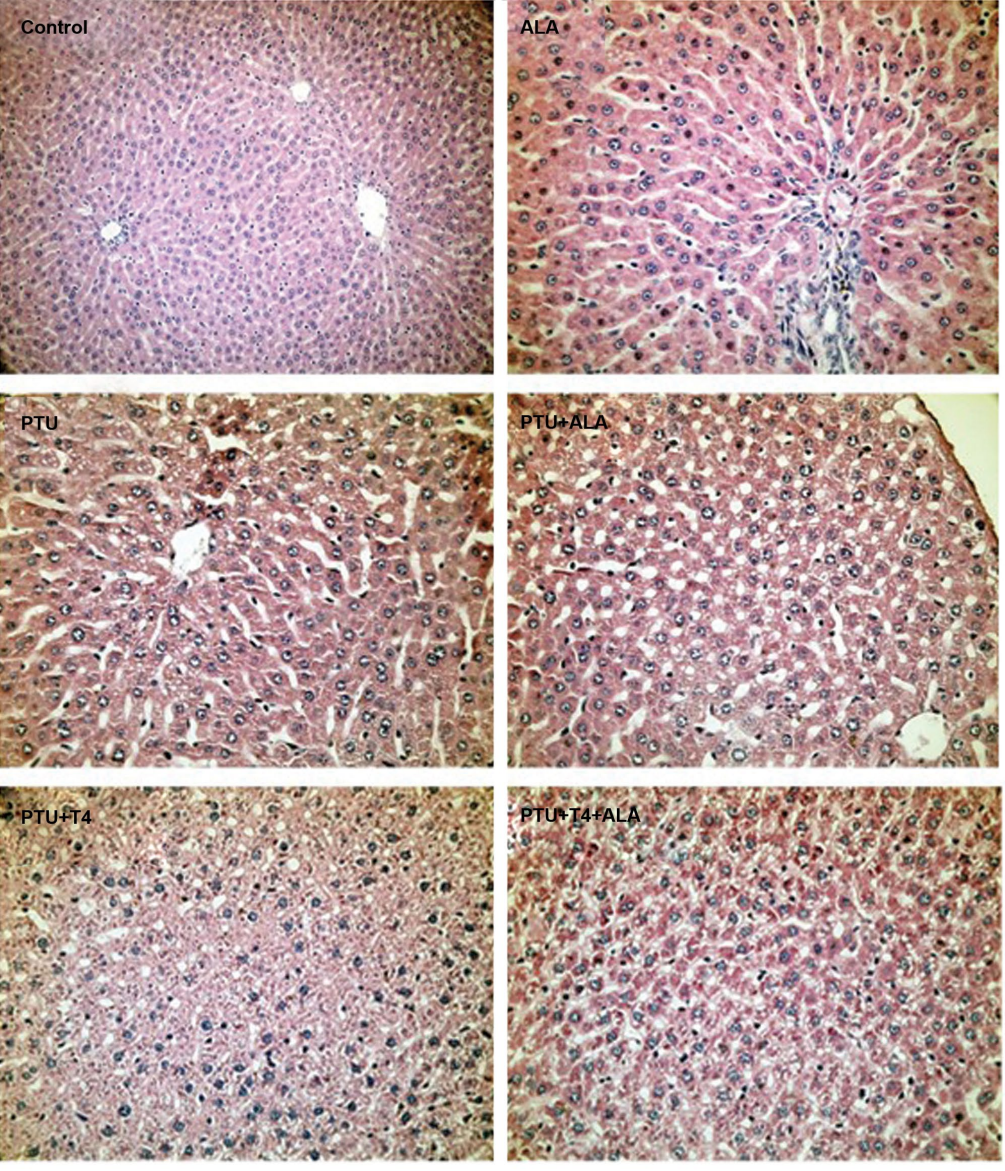
Histopathological findings in liver tissue of propylthiouracil (PTU)- administered rats treated by α-Lipoic acid (ALA) alone or together with T4 treatment (H&E, ×100).

The normal histological structure of the liver was observed in control and ALA groups. Hydropic degeneration around the central vein hepatocytes and congestion in sinusoids were observed in the PTU group. There were no changes in histopathological findings between PTU and PTU+ALA groups. However, necrotic and necrobiotic changes in hepatocytes and a decrease in sinusoid spaces were observed in the PTU+T4 group. Similar findings were also observed in the PTU+T4+ALA group, as observed in the PTU+T4 group (Fig .5). There was no difference in histopathological findings between the groups in brain tissue (data not shown).

## Discussion

Various methods are being used to create an experimental hypothyroid state, including thyroidectomy or drug administration, which suppresses TH synthesis such as PTU/methimazole (4, 9, 15, 26). It has become a matter of debate that methimazole is toxic to various tissues, and whether the effects are due to hypothyroidism or the toxic effect of methimazole (9). Similar to other antithyroid drugs, PTU decreases the synthesis of THs by suppressing thyroid peroxidase and prevents the conversion of T4 into T3 in peripheral tissues (4, 8).

In our study, hypothyroidism was reached by giving of PTU (500 mg/L) to rats in drinking water (4, 8). Indeed, a significant reduction in the levels of fT3 and fT4 was found in the serum of PTU-treated rats. There was a decrease in serum glucose and TG levels and an increase in cholesterol levels in hypothyroid rats. Although there were no differences in ALT and AST activities reflecting liver function, histopathological examination revealed hydropic degeneration around the central vein and constriction of the sinusoids in the liver. These findings are in accordance with previous studies (10, 26).

Many studies examined oxidative stress parameters in various tissues such as the liver, heart, brain, and kidney obtained from hypothyroid animals, such as rodents. Data obtained in these studies are controversial. Various factors such as type of antithyroid drugs used to induce hypothyroidism, duration of administration, the severity of the developed hypothyroid condition, different sensitivity of tissues to oxidative stress, and differences in the methods used to investigate prooxidant-antioxidant balance may explain these contradictory results.

In rat models of hypothyroidism, hepatic lipid and protein oxidation were found to be increased (7, 9, 26), not altered (27), and even decreased (6). Conflicting results have also been reported for antioxidant parameters (9, 27). Similar findings are also obtained in hypothyroid patients (11, 12). In this study, we found that ROS, MDA, and PC levels, together with antioxidant parameters, did not alter in the liver of PTU-induced hypothyroid rats. These findings indicate that hepatic prooxidant-antioxidant balance remained unchanged in hypothyroid rats.

On the other hand, THs possess important effects on brain development, maturation, and function. For this reason, the examination of brain tissue in hypothyroidism has gained particular importance (4, 14, 15). In many studies, increased lipid and protein oxidation products and weakness of the antioxidant system were observed in the total brain or specific brain regions in adult rats (4, 14), although some conflicting results are available (15). In the current study, brain MDA and PC levels were also observed to increase, but GSH levels and SOD activity decreased in PTU-treated rats.

It has been suggested that antioxidant treatment may be useful in the prevention of oxidative stress seen in hypothyroid state and may provide support to conventional treatments. ALA is a disulfide compound naturally found in mitochondria. Exogenous supplemented ALA can act as a powerful antioxidant which alleviates oxidative stress (17, 28, 29). Because ALA is a small lipophilic molecule, it easily crosses biological membranes, thus reaching all cellular compartments. Several studies have also shown that ALA is useful in the amelioration of various oxidative stress-induced pathologies, including atherosclerosis, metabolic syndrome, diabetes mellitus (13, 28). It was reported that ALA administration decreases obesity, thereby restoring blood TH levels and alleviating oxidative stress in the rats with long-term obesity (28). However, there are only two studies about the effects of ALA in hypothyroidism (29, 30). In one clinical study, 300 mg/ day ALA was administered for 3 weeks to patients with subclinical hypothyroidism, and an ameliorative effect on endothelial dysfunction together with decreased ROS was observed (29). Also, Tanaka et al. reported that in PTUexposed offspring, postweaning exposure to ALA may be efficient for improving developmental hypothyroidisminduced disruptive neurogenesis (30).

In our study, ALA was given during the last 5 weeks of PTU treatment. The application of ALA did not affect the observed changes in serum THs levels and examined biochemical parameters except ALT activity in the PTU group. Interestingly, there was an increase in serum ALT levels. Some investigators have suggested that ALA may have a toxic effect on the liver, depending on the ALA dose and duration of administration. However, no difference in the hepatic histopathological findings was found in the PTU and PTU+ALA groups. In addition, although ROS levels were detected to decrease, there were no changes in other hepatic oxidative stress parameters between PTU and PTU+ALA groups. On the contrary, in brain tissue, ROS, MDA, and PC levels decreased, but GSH levels increased in PTU-treated rats due to ALA treatment. These results indicate that ALA treatment was effective to decrease PTU-induced oxidative stress in brain tissue.

In this study, T4 replacement therapy was also applied in hypothyroid rats. However, after this treatment, serum T4 levels were found to be significantly increased when compared with both PTU and control values. This hyperthyroid condition created a change in the direction of prooxidation in the prooxidant-antioxidant balance in the liver tissue, together with increased serum AST activity and hepatic histopathological changes such as necrosis and decrease in sinusoidal spaces were observed. However, when ALA was administered to rats in the PTU+T4 group, serum AST activity decreased, but there were no differences in hepatic histopathological findings. Also, ALA administration was observed to cause a tendency to ameliorate changes in hepatic prooxidantantioxidant balance observed in PTU+T4 rats.

However, brain MDA levels increased, but ROS and PC levels did not alter in the brain tissue of rats of the PTU group due to T4 treatment. Additionally, significant increases in brain GSH levels and SOD activity were found in PTU+T4-treated rats. These increases may be an adaptive increase against the oxidative stress observed in brain tissue, and this situation may be a preventive factor against the development of a more intense pro-oxidant environment in brain tissue in PTU-treated rats due to T4 administration. Indeed, no differences in histopathological findings were also observed in brain tissue. When ALA was administered to rats in the PTU+T4 group, oxidative stress parameters and histopathological findings were found to not change in brain tissue.

## Conclusion

The susceptibility of liver and brain tissues to oxidative stress appears to be different, and the prooxidant state developed mainly in the brain of PTU-induced hypothyroid rats. ALA treatment has an improving effect on brain oxidative stress. In addition, ALA was also observed to be effective against hepatic oxidative stress arising from T4 replacement therapy. Our results indicate that ALA alone and together with T4 may be useful in reducing oxidative stress in thyroid dysfunctions.
